# The Views of Hospital Secretaries

**Published:** 1911-06-10

**Authors:** 


### THE VIEWS OF HOSPITAL SECRETARIES.

### Some Points from West Ham.

Mr. Thomas Cook, Chairman of the General Committee of Management of the West Ham and Eastern General Hospital, whose article on the effect of State insurance on hospitals we published last week, has kindly forwarded us the following addendum to his original article:—

With regard to the State Insurance Bill and the effect on Voluntary Hospitals, I have ascertained from the Secretary of the West Ham and Eastern General Hospital the following figures, comparing January 1 to May 30, 1910, with the statistics of the period January 1 to May 31, 1911: 1910.—All classes of patients, 13,958; casualties, 5,695; total attendances, 43,897. 1911.—All classes of patients, 14,705; casualties, 5,868; total attendances, 46,235. From which you will see what a heavy increase we have had in attendances of all classes of patients. He further informed me that 107 cases were admitted last month as in-patients. When I tell you that we have only 100 beds, and that last year the average length of time occupied per bed per in-patient amounted to 29.27 days, you will realise what a heavy admission this is for a month, for a hospital with 100 beds.

In chatting the matter of the State Insurance Bill over with the secretary, we came to the unanimous conclusion that if the Bill is passed into law in its present state the following is not an unintelligent anticipation:

Dealing with subscribers in three classes—the employer, the private individual, and the worker.

(1) We cannot but anticipate that the employer will subscribe less to voluntary institutions, knowing that his workers are provided for by themselves, the State and himself.

(2) The private individual feeling that he, as taxpayer, is providing for the workers and having to put his hand in his pocket for his domestic servants, will probably reduce some of his charitable subscriptions.

(3) The worker, having to pay weekly for health benefit, will probably reduce some of his charitable contributions, and under this heading we feel sure that the Sunday and Saturday Hospital Funds (probably the latter more than the former) will be the worse sufferers.

With regard to admissions: From the figures given above, you will see that the tendency of casualties is to increase, and we see no reason why the Bill should cause any change in this tendency.

The Bill does not provide any sick assistance for the wives and families of the workers, other than in maternity cases, with which class of patient our hospital does not deal, so we cannot expect any serious diminution in the number of out-patients' attendances or admissions as in-patients.

Thus we are faced with the problem of growing expenses and probably diminished receipts in a district where the population has gone up more rapidly than probably any other district in the United Kingdom. West Ham itself has a population of 360,000, and the surrounding district, from which we get abundant cases admitted to our hospital, has a total number of inhabitants which is estimated at somewhat over a million. We are practically the only General Hospital serving this huge district. The nearest other General Hospital—the London—is more than three miles away.—Yours faithfully,

Thos. Alex Cook,
Chairman of the General Committee of Management, West Ham Hospital.

### The Club Doctor and the Insurance Scheme.

The current number of *The Prescriber* contains a thoughtful note on one phase of the opposition to the Bill on the part of the medical men in this country:—

Under the proposals now before Parliament the genus club doctor is to be enlarged to a national magnitude without any hope that its natural defects will cease to exist. When the greater part of forty millions of people receive their medical treatment free through Friendly Societies, the individual interest of the doctor in his patient will cease; the patient will be deprived of the privilege of choosing his own doctor, and the present position of the physician as the friend and confidant of the family will no longer obtain. That wholesome competition which develops individuality, and makes for progress in medicine as in all walks of life, must of necessity cease with the nationalisation of the physician, and there will be the risk of scientific progress in medicine suffering from the inevitable lack of emulation and personal ambition. And not merely will the practice of medicine be nationalised, and, as we submit, emasculated, but the saving grace of individualism in the patient will be eliminated. He becomes a mere ticket-holder; the relation between doctor and patient is no longer a personal one. Moreover, one of the strongest incentives to clean and healthy living is dread of the material loss incident to illness. With that dread removed, the tendency, especially among the ignorant classes, will be towards a less careful method of living, a tendency that cannot be conducive to national health. Indeed, it is more than likely that the working of the scheme will result in a material increase of minor ailments among the community.

With these reflections most medical men will be inclined to agree, though we think that our contemporary makes too much of the final argument.

